# Dietary Neurotransmitters: A Narrative Review on Current Knowledge

**DOI:** 10.3390/nu10050591

**Published:** 2018-05-13

**Authors:** Matteo Briguglio, Bernardo Dell’Osso, Giancarlo Panzica, Antonio Malgaroli, Giuseppe Banfi, Carlotta Zanaboni Dina, Roberta Galentino, Mauro Porta

**Affiliations:** 1 Tourette’s Syndrome and Movement Disorders Centre, I.R.C.C.S. Galeazzi Hospital, 20161 Milan, Italy; carlotta.zanaboni@libero.it (C.Z.D.); roberta.galentino@gmail.com (R.G.); mauroportamilano@gmail.com (M.P.); 2 Department of Pathophysiology and Transplantation, I.R.C.C.S. Ca’ Granda Foundation, Ospedale Maggiore Policlinico, 20122 Milan, Italy; bernardo.dellosso@policlinico.mi.it; 3 Department of Psychiatry and Behavioral Sciences, School of Medicine, Stanford University, Stanford, CA 94305, USA; 4 Department of Neuroscience, Rita Levi Montalcini, University of Turin, 10126 Turin, Italy; giancarlo.panzica@unito.it; 5 Neurobiology of Learning Unit, Division of Neuroscience, Vita-Salute San Raffaele University, 20132 Milan, Italy; malgaroli.antonio@unisr.it; 6 Scientific Direction, I.R.C.C.S. Galeazzi Hospital, 20161 Milan, Italy; banfi.giuseppe@fondazionesanraffaele.it

**Keywords:** functional foods, neurotransmitters, diet, food, and nutrition, acetylcholine, glutamate, gamma-aminobutyric acid, dopamine, serotonin, histamine, gut microbiota

## Abstract

Foods are natural sources of substances that may exert crucial effects on the nervous system in humans. Some of these substances are the neurotransmitters (NTs) acetylcholine (ACh), the modified amino acids glutamate and γ-aminobutyric acid (GABA), and the biogenic amines dopamine, serotonin (5-HT), and histamine. In neuropsychiatry, progressive integration of dietary approaches in clinical routine made it necessary to discern the more about some of these dietary NTs. Relevant books and literature from PubMed and Scopus databases were searched for data on food sources of Ach, glutamate, GABA, dopamine, 5-HT, and histamine. Different animal foods, fruits, edible plants, roots, and botanicals were reported to contain NTs. These substances can either be naturally present, as part of essential metabolic processes and ecological interactions, or derive from controlled/uncontrolled food technology processes. Ripening time, methods of preservation and cooking, and microbial activity further contributes to NTs. Moreover, gut microbiota are considerable sources of NTs. However, the significance of dietary NTs intake needs to be further investigated as there are no significant data on their bioavailability, neuronal/non neuronal effects, or clinical implications. Evidence-based interventions studies should be encouraged.

## 1. Introduction

Diet, dietary modifications, and dietary supplements are identified as Complementary and Alternative Medicine (CAM) approaches, as they can either integrate or replace conventional therapies. In the current medical panorama, traditional medicine branches, such as neurology and psychiatry, evidenced an increasing incorporation of CAMs. Health professionals started to integrate dietary modifications for some neurological conditions, such as headaches [1], with positive results. Other conditions may directly (for example, drug-resistant epilepsy) [2] or indirectly (for example, Parkinson’s disease drug therapy) [3] depend on CAMs for their clinical outcome. Beyond their medical use, CAMs are often misused by patients [4], thus, possibly causing adverse effects, drug interactions, and a needless waste of money [5]. The large consumption of CAMs in neuropsychiatric patients is mainly due to the beliefs that food can be a mood modulator. Indeed, food has been recognized to affect mood depending on the availability of neurotransmitter (NT) precursors [6] and recompensing mechanisms [7]. Moreover, dietary supplements of certain micronutrients might help patients to recover from lethargy and depression, respectively, with iron and folate integration [8]. Despite the growing clinical attention to the effects of food on the nervous system, there is insufficient data on NTs food sources. Common NTs in humans, such as acetylcholine (ACh), the modified amino acids glutamate and γ-aminobutyric acid (GABA), and biogenic amines like dopamine (DA), serotonin (5-HT), and the well-known histamine (His), are also found in some animal foods, fruits, edible plants, and roots [9]. Despite plants and animals belonging to different kingdoms, chemical structures of NTs found in both of them are comparable. ACh, GABA, and 5-HT may naturally occur as primary or secondary metabolic products, respectively participating in essential metabolic processes or as a result of ecological interactions. Furthermore, other biogenic amines and glutamate can be products of microbial processing, food technologies [10], and voluntary additions. The current paper aims to review data from relevant books and PubMed/Scopus databases concerning NT food content, with a particular focus on ACh, glutamate, GABA, dopamine, 5-HT, and histamine.

## 2. Overview of Dietary Neurotransmitters

### 2.1. Acetylcholine

**Figure 1:**
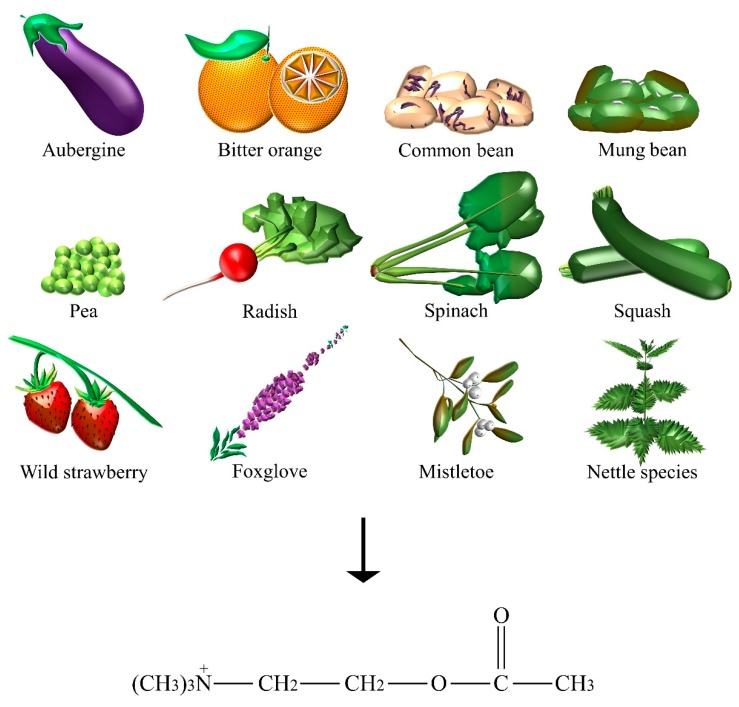
The dietary sources of acetylcholine.

In humans, ACh serves as a NT at the neuromuscular junctions, ganglionic synapses, and at diverse sites within the central nervous system. Its presence (Figure 1) is documented in more than 50 plant species belonging to all the major systematic groups, comprising the most economically important plant families: Gramineae, Leguminosae, and Solanaceae [11]. In particular, extracts from *Cucurbita pepo* L. (that is, squash), *Solanum melongena* L. (that is, aubergine), and *Spinacia oleracea* L. (that is, spinach) were reported to contain a considerable amount of ACh [12]. Besides its presence in plants that could suggest its role in the regulation of membrane permeability or specific metabolic pathways [13], ACh was found in the seeds of *Pisum sativum* L. (that is, pea), *Phaseolus radiatus* L. (that is, mung beans), and *Phaseolus vulgaris* L. (that is, common bean), thus, indicating a possible role during germination [11]. The fruits of *Citrus aurantium* L. (that is, bitter orange), *Fragaria vesca* L. (that is, wild strawberry) [14], and the edible root vegetable of *Raphanus raphanistrum* subspecies *sativus* L. (that is, radish) [15] were indicated to contain Ach. The highest concentrations of ACh was found in the nettle species of *Urtica dioica* L. (for example, about 0.5 μmol/g dry weight of roots) [14] and of *Urtica ureus* L. [16], whose folium and herba are traditionally used as adjuvants in minor urinary problems and articular pain. Other plants, such as *Viscum album* L. (that is, mistletoe) and *Digitalis purpurea* L. (that is, foxglove), contain significant amounts of ACh [11]. In particular, mistletoe had a traditional use in the treatment of patients with high blood pressure, arteriosclerosis, hypertensive headache, epilepsy, chorea, hysteria, and other neurological diseases [17]. The cardiac-depressant and sedative properties of mistletoe were attributed to various biologically active constituents, such as ACh itself, but also to histamine and GABA [18].

### 2.2. Glutamate

**Figure 2:**
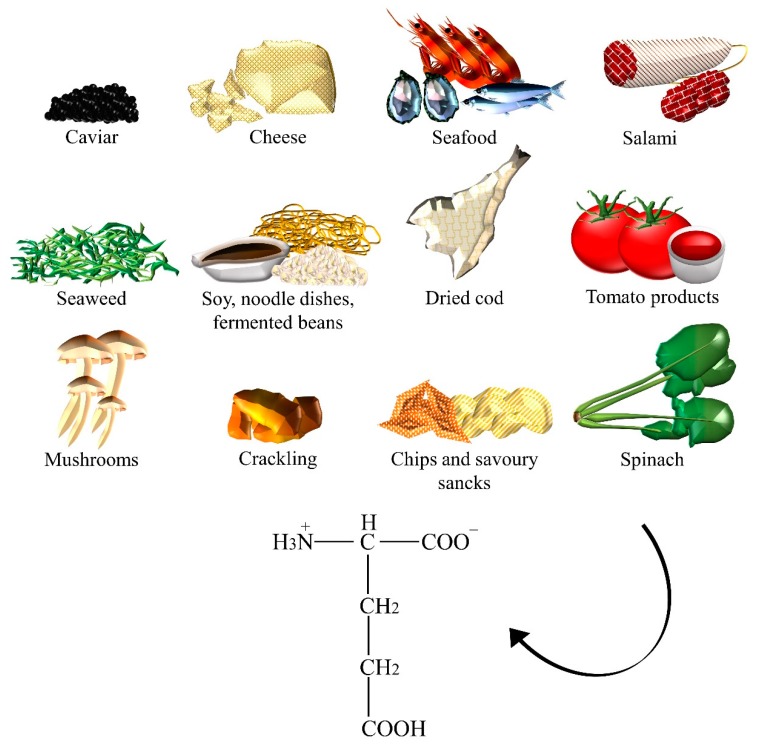
The dietary sources of glutamate.

In humans, glutamate is a non-essential amino acid and the most important excitatory NT in the brain. Glutamate (Figure 2) and glutamic acid are ubiquitously present in foods. At pH 7, dietary glutamic acid is transformed into glutamate, which is its anionic form. Glutamic acid naturally occurs in foods with high protein content (for example, meats, seafood, stews, soups, and sauces) [19]. Seaweeds, cheeses, fish sauces, soy sauces, fermented beans, and *Solanum lycopersicum* L. (that is, tomato) showed high levels of free glutamic acid [20]. Dried cod, cracklings, salami, caviar, and instant coffee powder are other well-known sources of this amino acid. Salts of glutamic acid, such as sodium, potassium, calcium, and magnesium, can be added to certain foods or sauces as flavor enhancers [21]. Upon ingestion, monosodium glutamate and other glutamate salts dissociate, releasing free glutamate. Foods sources of monosodium glutamate and glutamic acid are often the same: fish sauces, oyster sauce, tomato sauce, gravies, miso, noodle dishes, Parmesan cheese, savoury snacks, chips, ready-to-eat meals, but also, mushrooms and spinach [22].

### 2.3. Gamma-Aminobutyric Acid

**Figure 3:**
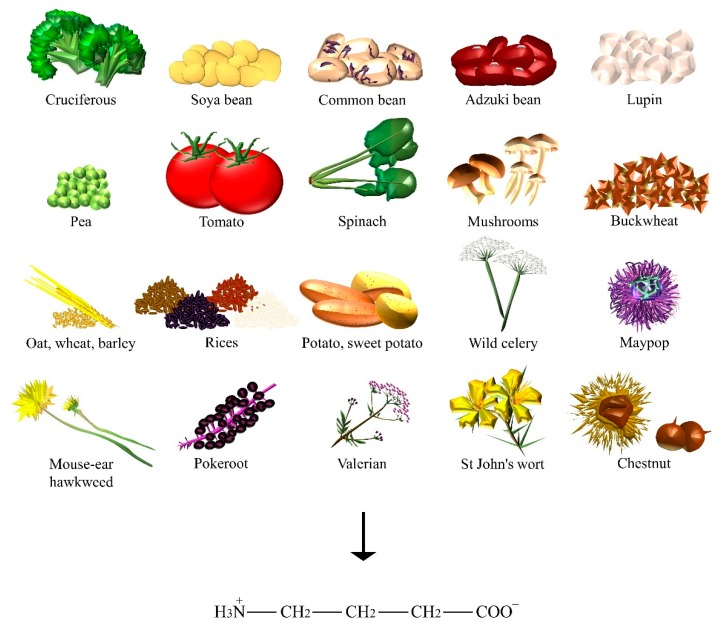
The dietary sources of GABA.

GABA is a major inhibitory NT of the vertebrate central nervous system and is found ubiquitously among plants (Figure 3), where can be primarily synthesized from glutamic acid via glutamate decarboxylase enzyme. Levels of GABA were demonstrated to increase in response to biotic and abiotic stresses, such as drought, the presence of salt, wounds, hypoxia, infection, soaking, and germination [23]. In particular, sprouts of *Lupinus angustifolius* L. (that is, lupin) [24], *Vigna angularis* W. (that is, adzuki bean) [25] and other germinating edible beans, such as *Glycine max* L. (that is, soya bean) [26], common bean, and pea [27], were reported to increase GABA content when compared to their raw beans. Furthermore, grains of the *Gramineae* family, such as *Avena nuda* L. (that is, oat) [28], *Triticum aestivum* L. (that is, wheat) [29], *Hordeum vulgare* L. (that is, barley) [30], and many species of the *Oryza* genus (for example, white, black, brown, and red rice) [23] can also significantly accumulate GABA. Sprouts of *Fagopyrum esculentum* M. (that is, buckwheat) [31] and the fruits of tomato also contain a substantial amount of this amino acid during the mature green stage [32]. GABA is known for its analgesic effects, anti-anxiety, and hypotensive activity. Food technologies and molecular engineering are employed to synthesize GABA through enzymatic or whole-cell biocatalysis, microbial fermentation (for example, GABA soya yogurt [33], black raspberry juice [34]), and chemical synthesis [35]. Some authors found one of the highest contents on GABA to be 414 nmol/g of dry weight in raw spinach, followed by *Solanum tuberosum* L. (that is, potato), *Ipomoea batatas* L. (that is, sweet potato), and *Brassica oleracea* L. (that is, cruciferous such as kale and broccoli). Mushrooms, such as *Lentinula edodes* B. (that is, shiitake), and nuts of *Castanea* genus (that is, chestnut) also showed a significant amount of GABA [30]. Among the many types of Chinese teas, the highest content was found in white tea [36]. As already mentioned, GABA content was found in mistletoe [18], but also in *Phytolacca americana* L. (that is, pokeroot) [37], *Valeriana officinalis* L. (that is, valerian), *Angelica archangelica* L.(that is, wild celery), *Hypericum perforatum* L. (that is, St John’s wort), *Hieracium pilosella* L. (that is, mouse-ear hawkweed), and *Passiflora incarnata* L. (that is, maypop) [38], the latter being used for the relief of mild symptoms of mental stress and as a sleep aid.

### 2.4. Dopamine

**Figure 4:**
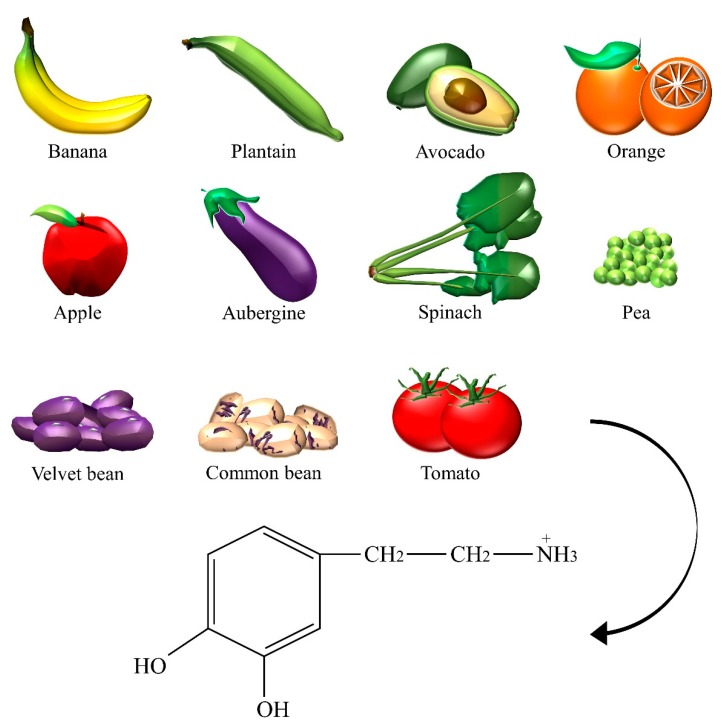
The dietary sources of dopamine.

Dopamine plays an essential role in humans for the coordination of body movements, motivation, and reward. Information regarding the content of dopamine foods (Figure 4) is very limited, possibly because of lack of clinical interest. Fruits of the *Musa* genus, such as bananas and plantains, and the *Persea americana* M. species (that is, avocado) were reported to contain high levels of dopamine [39]. More specifically, dopamine levels were found in the banana peel (700 μg/g), the banana pulp (8 μg/g), and in avocado (4–5 μg/g). In plants, dopamine exerts a protective role and is involved in reproductive organogenesis, ion permeability [11], antioxidant activity [40], and in the formation of alkaloids [41]. Interestingly, leaves of *Mucuna pruriens* L. (that is, velvet bean) were proven to contain dopamine [42], thus, being possibly involved in the well-known anti-parkinsonian effects of the products obtained from the seeds [43]. Low levels were measured in *Citrus sinensis* L. (that is, orange), *Malus sylvestris* L. (that is, forest apple), tomato, aubergine, spinach, pea, and the common bean [39]. Episodic movement disorders (that is, shaking the head from side to side) were reported after the consumption of skim milk. Same authors attributed these effects to the high content of L-tyrosine in dairy products [44]. However, a possible interaction of dopamine is not to be excluded, but the literature data is insufficient.

### 2.5. Serotonin

**Figure 5:**
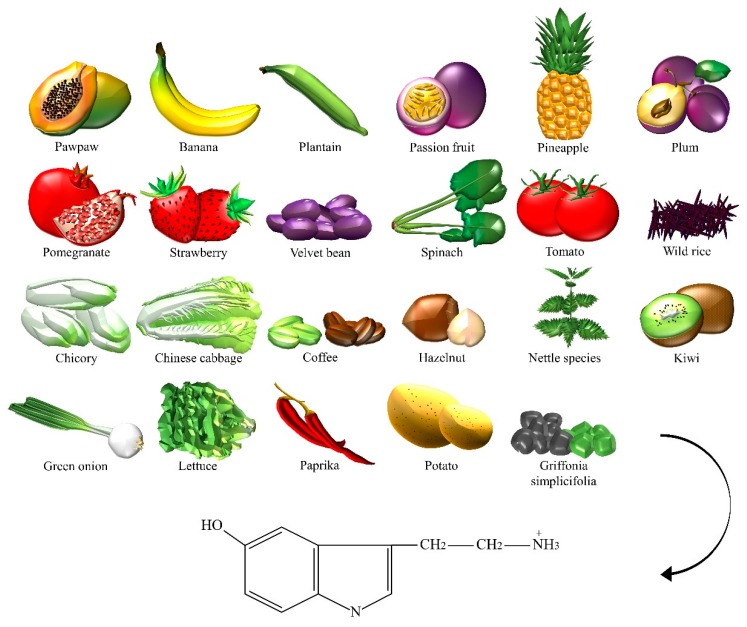
The dietary sources of serotonin.

In the central nervous system, 5-HT pathways modulate behaviors, eating, and sleep, whereas, in the gut, they are involved in the regulation of gastrointestinal motility. Fruits, vegetables, and seeds are major sources of 5-HT (Figure 5). In recent years, the number of studies on the content of 5-HT in plants has increased, greatly encouraged by the discovery of melatonin, which stimulates the late vegetative growth of different tissue sections [45]. 5-HT appeared to be prevalent in the green fruit of the *Musa* genus (that is, prata banana, and other species), containing about 7100–21,000 ng/g of fresh weight, followed by a significant decrease during ripening [46]. Higher concentrations were found in banana peels compared to the pulp [41]. The accumulation of 5-HT was also detected in *Capsicum annuum* L. (that is, pepper) [47], and paprika [48]. 5-HT was identified in *Corylus avellana* L. (that is, hazelnut) [49], fruits of tomato and cherry tomato [48], *Ananas comosus* L. (that is, pineapple) [50], *Prunus domestica* L. (that is, plum) [41], *Passiflora edulis* S. (that is, passion fruit), *Carica papaya* L. (that is, pawpaw) [51], and in fruits of the *Actinidia* genus (that is, kiwi) [52]. Similar to dopamine, 5-HT was found in the velvet bean [53]. The authors detected about 34,400 ng/g of dry weight in spinach [48]. *Brassica rapa* L. (that is, Chinese cabbage) [48], potato leaves [54], rice plant, and seeds of *Oryza sativa* L. (that is, wild rice) [55], were also considered sources of 5-HT. This NT was found in green coffee beans and, because of its high resistance to roasting, even in coffee powders [56]. Traces were found in *Punica granatum* L. (that is, pomegranate), fruits of the *Fragaria* genus (that is, strawberry) [57], *Cichorium intybus* L. (that is, chicory), *Allium ascalonicum* L. (that is, green onion), and *Lactuca sativa* L. (that is, lettuce) [48]. Some plants, such as nettle [58] and *Griffonia simplicifolia* DC were found to contain 5-HT. *Griffonia* was marketed for its presumptive anxiolytic effects that were later associated with the content of 5-hydroxy-l-tryptophan, a direct precursor in the synthesis of serotonin [59].

### 2.6. Histamine

**Figure 6:**
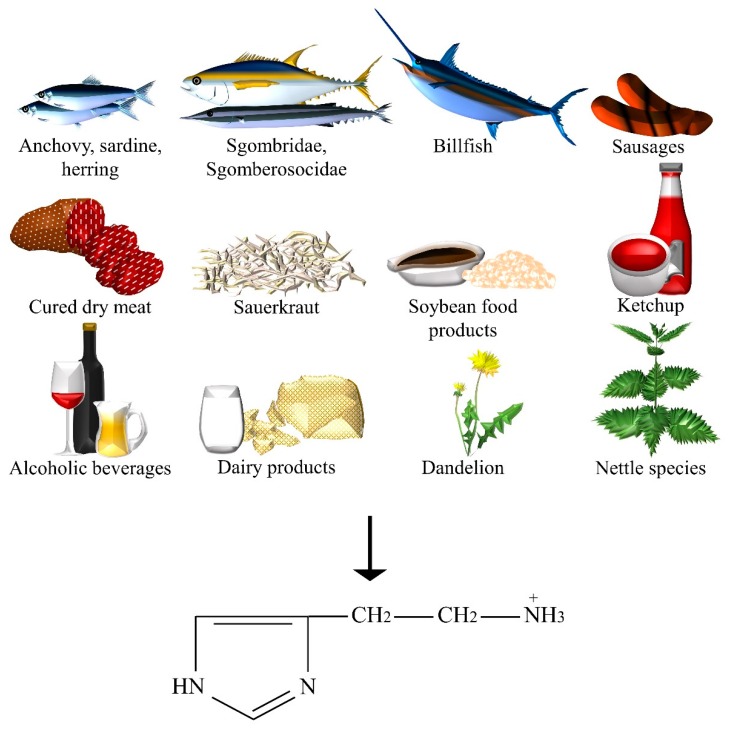
The dietary sources of histamine.

Histamine is an NT that is present in mammalian hypothalamic neurons with widespread projections to nearly all regions of the brain mediating arousal, attention, and reactivity. It is a heterocyclic, nitrogenous, and naturally occurring compound formed from histidine (Figure 6). Despite being considered endogenous in certain foods, relatively high levels of histamine and other biogenic amines indicate defective food processing, microbial activity, and general deterioration. In fact, the food industry aims to maintain the levels of amines in foods as low as possible in order to meet the quality standards. Consumption of fish, ham, and other cured dry meat products [60], sauerkraut, and cheese varieties such as Cheddar, Swiss, Gruyère, and Gouda were associated with amine poisoning [61]. The release of adrenaline and noradrenaline, the excitation of smooth muscles within intestines and respiratory tract, the stimulation of both sensory and motor neurons, and the excessive gastric acid secretion were associated with histamine intoxication [61]. The presence of histamine in the sting of the nettle can cause hives and edema [62]. On the other hand, the presence of histamine in processed foods, such as aged cheeses, is necessary to achieve characteristic flavors and textures. Red, white, dessert wines, Champagne, Sherry [63], and possibly beer may contain a significant amount of histamine. In addition, fish could be a food source of histamine, depending on its exposure to microbial contamination or unfavorable storage conditions. Poisoning could result from the consumption of fishes belonging to the families of Scombridae (for example, tuna, mackerels, and bonitos), Scomberesocidae (for example, sauries), and others, such as sardine, anchovies, herring, and billfishes [61]. Dairy products were identified with a significant amount of histamine: cheese contains up to 2500 ppm, followed by yogurt, sweet or sour cream, UHT milk, pasteurized milk, and fresh milk [64]. Histamine was also detected in fermented sausages [65], ketchup [66], and soybean products, including fermented soy, tempeh, soy sauce, soya bean milk, soybean paste (that is, doenjang), doufuru (that is, salted and aged tofu), and nattō [67]. Then, among plants, *Taraxacum officinale* L. (that is, dandelion) presented high levels of histamine, as well as many other herbs whose pollens are used in phytotherapy [68].

### 2.7. What about Microorganism-Derived NTs?

Many other factors could contribute to dietary NTs availability: ripening time, preservation methods, cooking methods, and microbial activity (for example, the formation of biogenic amines) [10]. Bad manufacturing practices may lead to the contamination with pathogenic bacteria or fungi (for example, *Bacillus subtilis*) [69]. Other symbionts in the human gastrointestinal tract were shown to actively contribute to the production of the aforementioned NTs, thus, possibly exerting effects on the nervous system. *Lactobacillus* species were demonstrated to produce ACh [70]. *Lactobacillus brevis* and *Bifidobacterium dentium*, found in the human intestines, were able to produce GABA from monosodium glutamate, with up to almost a 100% conversion efficiency for a specific strain of *Lactobacillus* [71]. *Lactococcus* species showed considerable GABA production capacity [72] and GABA-producing strains were isolated from Italian cheese [73], whole milk [74], and commercial soy sauce [75]. Among fungi, *Aspergillus nidulans* might play a role in GABA production [76]. Members of the *Bacillus* and *Serratia* genera were said to play a crucial role in generating the biologically active dopamine in the lumen of the gut [70,77]. Serotonin-producing bacterial strains were identified as belonging to *Lactococcus lactis* species, *Lactobacillus plantarum*, *Streptococcus thermophiles*, *Escherichia* species, *Morganella morganii*, *Klebsiella pneumoniae*, *Hafnia alvei* [78], *Candida* species, and *Enterococcus* species [79]. Regarding histamine, *Lactobacillus reuteri* can exert luminal conversion of ingested histidine to histamine [80], but potentially many others [81]. In conclusion, a considerable exogenous source of NTs could be gut microbiota, thus, the designation of psychobiotics appears to be established [82]. Furthermore, food is a direct modulator of gut microbiota, eventually establishing a *ménage à trois* among the diet, gut microbiota, and brain. These relations are even more complex considering that hormones, in particular steroid hormones, can influence the gut microbiota and, in turn, the gut microbiota can influence circulating hormone levels [83]. Furthermore, some extensive contaminants, such as the endocrine disruptors, may influence the gut bacteria composition [84].

## 3. Discussion

All foods that were reported to contain different NTs are summarized in Table 1. The fact that different quantitative and qualitative analytical methods were used for determining NTs content in foods represents the main limitation. Animal (for example, fish) and processed foods (for example, wine) might show little variability, while, in plants, there are many issues to be considered. Parts of the same plant, such as the stem, leaves, inflorescence, flowers, and fruits may have different properties and contain different levels of NTs. As for nutrients and nutraceuticals, dietary NT contents vary according to the subspecies and varieties of plants (that is, morphological differences), cultivar and ecotype (that is, diversity of environmental adaptation), chemotype (that is, different molecular profile), soil and nourishment, geographical location, environmental impact during plant growth, seasons of growth and harvest, weather and climate changes, and agricultural practices.

Once ingested, dietary NTs may exhibit possible roles in non-neuronal tissues, such as for ACh, dopamine [16,85], and 5-HT, which are known to be involved in gastrointestinal motility. For instance, one of the evolutionary roles of phytoserotonin is to ensure that the seeds of endozoochorial plants are evacuated, by activating enteric neurons involved in the migrating motor complexes in the colon [86]. Conversely, there may be some inactivating enzymes that limit the effects of dietary NTs, as the erythrocytes and blood acetylcholinesterases do for ACh [16], thus, guaranteeing a functional separation between the role of local mediators and NTs. As for glutamate, the major part of 5-HT typically undergoes extensive intestinal metabolism. Barriers usually prevent direct passage of xenobiotic substances from the blood to the brain, but both histamine and 5-HT showed the ability to increase the permeability of the blood-brain barrier [87]. More specifically, 5-HT can cross the barrier either from the brain to blood [88] or possibly vice versa through transporters that were found in the largest pial vessels and smallest brain capillaries of adult rats [89]. Points of communication between the blood, brain parenchyma, and central nervous system exist, such as the circumventricular organs (for example, median eminence, the subfornical organ, and the area postrema) that allow the access of circulating cytokines, immunoglobulins, infectious agents, and proteins, leading to the possible exposure of local neurons, glial, and endothelial cells to these signals [90,91]. If these molecules had a unique role in the gut, it would be worth investigating their luminal or barrier disruptive effects, as food allergens were showed to have [92]. The translocation of a dietary NT into the bloodstream should be verified by measuring the blood concentration after ingestion and further considering its rate of elimination. Remarkably, a consistent arterial-venous increase in dopamine plasma levels was shown, thus, suggesting the presence of specific transporters and a consistent dopamine production in the gastrointestinal tract [85].

Few clinical evidence explored the role of dietary NTs on the nervous system:

- Monosodium glutamate was hypothesized to be associated with the Chinese restaurant syndrome [93] (for example, numbness, weakness, and heart palpitations), but also with vertigo [94]. However, the lack of significant evidence [95] may be explained by the fact that trials always enrolled patients with no particular sensitivity to glutamate, while positive cases reported in the literature referred to fragile or glutamate-sensitive individuals [96]. Dietary glutamate could exert central nervous system effects only during neonatal development: a subcutaneous injection of monosodium glutamate caused adult mice to be more prone to anxiety and depression-like behaviors [97]. Actually, facilitative glutamate transporters through the blood-brain barrier were found only on the luminal membrane [98].
- GABA was proven to have central nervous system action after an oral administration of 800 mg by modulating fronto-striatal networks [35]. Moreover, the benefit from the consumption of GABA-containing vegetables showed the importance of dietary GABA on the sympathetic nerve activity [99]. Conversely, there is still discordance over the alleged GABA capacity to cross the blood-brain barrier [100].
- The increase of the histamine concentration in the plasma was shown to be due to both the consumption of specific foods rich in histamine and to foods with histamine-releasing capacities (for example, citrus fruit, tomatoes). The positive results were demonstrated by diets with low histamine levels [101]. The intestinal diamine oxidase (DAO) enzyme normally prevent dietary histamine uptake into the blood circulation. However, excessive ingestion, the use of DAO inhibitor drugs, alcohol consumption, and cases of concomitant gastrointestinal diseases (for example, gastritis, irritable bowel syndrome, Crohn’s disease, and ulcers) or enzyme-deficiency, may compromise the catabolic capacity of this enzyme.

## 4. Conclusions

Macronutrients (for example, fats [102] and proteins [103]), micronutrients (for example, minerals [104] and vitamins [105]), but also non-nutrients (for example, nutraceuticals and alcohol) are topics of interest in neuropsychiatry. Recently, the nutritional side effects of drugs (for example, alteration of orexigenic/anorexigenic signals) and drug-nutraceutical interactions revealed new insights into the understanding of the interdisciplinary approach of nutritional neuroscience. From now on, psychobiotics, different animal foods, fruits, edible plants, roots, and botanicals will be seen as natural sources of neurotransmitters. Nonetheless, the significance of dietary NTs intake needs to be further investigated, as there are no significant data about their bioavailability or clinical implications. It is not unlikely that an adult nervous system can manage homeostatic alterations induced by dietary NTs. As molecular and neurobiological research progressively explains the etiopathogenesis of brain disorders, new studies should consider if these dietary NTs can escape gut microbiota metabolism, act on peripheral receptors, be transported across enterocytes, escape splanchnic metabolism, be transported across the blood-brain barriers (that is, capillary endothelium and choroid plexus epithelium) or exert central nervous system effects through circumventricular organs. In order to reasonably investigate the ability of dietary NTs to pass across the blood-brain barrier, both in vitro models and in vivo investigation should be performed to take into account the role of neuronal cells and the brain’s microvasculature [106]. If dietary NTs proved instead to be a central nervous system effect in thorough clinical/behavioral studies, foods and botanicals enlisted in Table 1 would be beneficial for subjects suffering from Alzheimer’s disease or dementia (for example, an ACh diet), epilepsy or migraines (for example, a glutamate-free diet), anxiety or insomnia (for example, a GABA diet), Parkinson’s disease (that is, a dopamine diet), depressive disorders (that is, a serotonin diet), and vascular headaches (that is, a histamine-free diet). Pragmatic approaches may be used for either augmented perceptions of stress or reduced mental outlook conditions as part of the nutritional psychiatry field [107]. Certainly, knowledge of these food sources could be a valuable starting point for anyone who seeks to investigate their potential effects on mental health, thus, being a possible hazard to fragile individuals [108,109] or during prenatal and early childhood development. A pragmatic approach to neuropsychiatric patients is necessary, possibly focusing on the implementation of CAMs in the conventional treatments. Yet, patients’ perceptions and expectations of some foods and supplements may be far from reality [110] and an accurate patient education should be provided. Knowledge of the presence of residues (for example, pesticides), additives (for example, colorants, artificial sweeteners), contaminants (microorganisms, heavy metals, endocrine disruptors, substances from food-packaging migration), adulterations with non-declared active ingredients, and of course dietary NTs, is the condition *sine qua non*, where there is no well aware nutrition.

## Figures and Tables

**Table 1:** The dietary neurotransmitters and relative food sources.

Dietary Neurotransmitter	Foods and Botanicals
Acetylcholine	Aubergine, bitter orange, common bean, foxglove, mistletoe, mung bean, nettle species, pea, radish, spinach, squash, wild strawberry.
Glutamate	Caviar, cheese, crackling, chips, dried cod, fermented beans, fish sauces, gravies, instant coffee powder, meats, miso, mushrooms, noodle dishes, oyster sauce, Parmesan cheese, ready-to-eat meals, salami, savory snacks, seafood, seaweeds, soups, soy sauces, spinach, stews, tomato, tomato sauce.
GABA	Adzuki bean, barley, broccoli, buckwheat, chestnut, common bean, kale, lupin, maypop, mouse-ear hawkweed, oat, pea, pokeroot, potato, rice, shiitake, soya bean, spinach, St John’s wort, sweet potato, tea, tomato, valerian, wheat, wild celery.
Dopamine	Aubergine, avocado, banana, common bean, apple, orange, pea, plantain, spinach, tomato, velvet bean.
Serotonin	Bananas, chicory, Chinese cabbage, coffee powders, green coffee bean, green onion, hazelnut, kiwi, lettuce, nettle, Griffonia simplicifolia, paprika, passion fruit, pawpaw, pepper, pineapple, plantain, plum, pomegranate, potato, spinach, strawberry, tomato, velvet bean, wild rice.
Histamine	Anchovy, beer, billfish, Champagne and Sherry, dandelion, fermented sausages, ham and other cured dry meat products, herring, ketchup, aged cheeses, nettle, red, white and dessert wines, sardine, sauerkraut, Scomberesocidae (for example, sauries), Scombridae (for example, tuna, mackerel, and bonitos), soybean food products (for example, soy, tempeh, soy sauce, soya bean milk, doenjang, doufuru, and nattō), sweet or sour cream, UHT, pasteurized, and fresh milk, yoghurt.
